# Comprehensive Review of Prevention and Management Strategies for Medication-related Osteonecrosis of the Jaw (MRONJ)

**DOI:** 10.3290/j.ohpd.c_2169

**Published:** 2025-08-05

**Authors:** Nasimeh Baghalipour, Omid Moztarzadeh, Walla Samar, Jiri Gencur, Vaclav Volf, Lukas Hauer

**Affiliations:** a Nasimeh Baghalipour Dentist and PhD Candidate, Department of Stomatology, University Hospital Pilsen, Faculty of Medicine in Pilsen, Charles University, Pilsen, Czech Republic. Database search, extracted data, wrote the manuscript.; b Omid Moztarzadeh Assistant Professor, Department of Stomatology and Department of Anatomy, University Hospital Pilsen, Faculty of Medicine in Pilsen, Charles University, Pilsen, Czech Republic. Idea, conceptualisation, supervision, wrote, reviewed and edited the manuscript.; c Walla Samara Dentist and PhD Candidate, Department of Stomatology, University Hospital Pilsen, Faculty of Medicine in Pilsen, Charles University, Pilsen, Czech Republic. Wrote, edited and reviewed the manuscript.; d Jiri Gencur Dentist and PhD Candidate, Department of Stomatology, University Hospital Pilsen, Faculty of Medicine in Pilsen, Charles University, Pilsen, Czech Republic. Assisted with literature organization, provided clinical input during the drafting and revision stages.; e Vaclav Volf Dentist and PhD Candidate, Department of Stomatology, University Hospital Pilsen, Faculty of Medicine in Pilsen, Charles University, Pilsen, Czech Republic. Wrote the manuscript.; f Lukas Hauer Associate Professor and Head, Department of Stomatology, University Hospital Pilsen, Faculty of Medicine in Pilsen, Charles University, Pilsen, Czech Republic. Senior author, supervised the overall manuscript development, provided critical revisions and oversight throughout the writing process.

**Keywords:** antiangiogenic, antiresorptive medication, cancer, medication-related osteonecrosis of the jaw, osteoporosis.

## Abstract

**Purpose:**

To assess and classify the strategies employed in various dental specialities for the prevention and management of medication-related osteonecrosis of the jaw (MRONJ).

**Materials and Methods:**

A comprehensive literature review was conducted, acquiring studies sourced from Google Scholar and PubMed. The emphasis was on studies published in recent years, focusing on successful MRONJ prevention techniques across various dental specialties.

**Results:**

Four types of prevention were identified. Primary prevention strategies include optimizing oral hygiene, managing dental caries, and extraction of hopeless teeth in patients before starting antiresorptive or antiangiogenic medications, to reduce MRONJ risk. Secondary prevention techniques involve tailored approaches during procedures employed during different dental specialties aimed at reducing complications in susceptible patients. Tertiary prevention focuses on managing established MRONJ, aiming to relieve symptoms and prevent further deterioration. Quaternary prevention seeks to limit overmedicalisation and reduce risks associated with medications that contribute to MRONJ development.

**Conclusion:**

Primary prevention remains the prevention of choice in terms of minimising the possible incidence of MRONJ, while secondary and tertiary prevention strategies are vital for managing risks and improving outcomes in susceptible patients. Quaternary prevention requires more research focusing on reducing the incidence of underlying conditions such as osteoporosis and cancer, which are associated with MRONJ development.

Osteoporosis, characterised by reduced bone density, often goes undiagnosed or untreated in many individuals, leading to increased bone fracture risk. In patients diagnosed with osteoporosis, antiresorptive (AR) drugs such as bisphosphonate and denosumab, are commonly used as part of treatment plans.^72^ While in patients with osteolytic bone diseases such as bone metastasis from solid tumors, treatment protocols typically include a combination of AR and antiangiogenic (AA) agents. These medications are essential for managing disease progression as well as skeletal consequences from osteoporosis or bone metastases, which lead to increased pain, mortality rates, and reduced quality of life.^46,50^ However, while these therapies are effective in controlling skeletal-related events, they may carry the risk of severe adverse effects, such as unusual femur fractures and osteonecrosis of the jaw.^72^ Hence, due to the increasing prevalence of MRONJ, dentists, oncologists, and physicians have to collaborate closely in a coordinated manner, while dentists may be in the first line to diagnose and treat patients, with a greater chance of success.^28,67,75^

In general, osteonecrosis in the jawbones can be classified broadly into osteoradionecrosis (ORN), medication-related osteonecrosis of the jaw (MRONJ), spontaneous osteonecrosis, non-traumatic osteonecrosis, and traumatic osteonecrosis.^40^ However, medication-related osteonecrosis of the jaw (MRONJ) has emerged as a distinct and growing clinical challenge. This review aims to provide a comprehensive analysis of MRONJ, with a specific focus on prevention strategies in different dental specialities, as well as the management of the condition once it has developed. To provide useful recommendations for dental practitioners who work with at-risk populations, this review addresses primary, secondary, tertiary, and quaternary prevention measures.

## MRONJ Definition

The American Association of Oral and Maxillofacial Surgeons (AAOMS) defines medication-related osteonecrosis of the jaw (MRONJ) as the exposure of the maxillary or mandibular bone that persists for more than eight weeks in a patient with a history of antiresorptive (AR) or antiangiogenic (AA) medications, often accompanied by concurrent immunosuppressant medications, and without any history of head and neck radiation therapy or evident metastatic disease to the jaws. MRONJ may present with an intraoral or extraoral fistula that probes to bone, or in some cases, without any visible fistula. It can occur spontaneously or following dental procedures, such as tooth extractions, ill-fitting dentures, or other invasive dental treatments.^58^

Although the main etiological factors for MRONJ are antiresorptive (AR) and antiangiogenic (AA) medications, new research indicates that additional drugs that are not typically associated with the condition may also play a role in its development. For example, MRONJ has been documented in patients treated with medications such as secukinumab for psoriasis,^29^ intravenous tocilizumab for rheumatoid arthritis,^62^ or long-term simvastatin for hypercholesterolemia.^63^

The jaw’s unique physiological and anatomical features, including its high intramembranous ossification, calcium levels, and collagen content, make it more susceptible to osteonecrosis.^30^ Ultimately, MRONJ is more prevalent among older women and predominantly affects the mandible (as compared to the maxilla).^33^

Although the exact pathogenesis of MRONJ is still unknown, two main explanations have been put forth:^42^

Inside-outside theory: Based on this theory, osteonecrosis starts internally, i.e., with bone inflammation, and processes externally with structural distortion, with antiresorptive agents inhibiting osteoclast activity and bone metabolism.^42^
Outside-inside theory: According to this view, bone necrosis develops from external sources, such as tooth or mucosal lesions, and advances through to the bone tissue.^42^

Understanding the theories of MRONJ pathogenesis is essential for identifying at-risk populations, who can be categorised based on medication use and other risk factors.

Patients at risk of MRONJ are generally divided into low-risk and high-risk groups. This classification is primarily based on the type, duration, frequency and administration method of medication used (Table 1).^36,58^ Notably, the International Task Force on ONJ 2016 reported that MRONJ occurs in approximately 0.001%—0.01% of osteoporosis patients treated with much lower doses of bisphosphonates or denosumab, while in contrast, the frequency is estimated at 1%—15% in oncology patients receiving higher doses of medications.^36^

**Table 1:** Patient risk-level classification and clinical decisions regarding MRONJ prevention

Patient group	Risk level	Characteristics	Examples
Osteoporosis patients	Low risk	Treated with antiresorptive medications (bisphosphonates or denosumab) for < 3 years No concurrent immunosuppressive medications	Osteoporosis patients taking Alendronate (oral bisphosphonates) Patients taking Prolia (Denosumab)
High risk	Treated with antiresorptive medications for > 3 years Treated with antiresorptive medications (bisphosphonates or denosumab) for < 3 years with concurrent use of immunosuppressive medications (e.g., steroids)	Osteoporosis patients with long-term treatment of Zoledronate (IV bisphosphonates) Long-term Denosumab intake Osteoporosis patients with short-term treatment using Zoledronate (IV bisphosphonates) with concurrent use of immunosuppressive medications (e.g., steroids)
Cancer patients	High risk	Treated with high-dose antiresorptive medications (IV bisphosphonates or denosumab) Concurrent use of antiangiogenic agents or chemotherapy Immunocompromised (e.g., multiple myeloma, metastatic cancers)	Cancer patients treated with Xgeva (Denosumab) or Zoledronate (IV bisphosphonates) Patients receiving Bevacizumab, Sunitinib (antiangiogenics)


Low-risk patients are those who have taken bisphosphonate or denosumab for treatment of bone diseases such as osteoporosis, without concurrent immunosuppressive medications or conditions, for less than three years.^58^

High-risk patients are those who have been taking bisphosphonate or denosumab for bone disease treatment for more than three years, or less than three years but with concurrent immunosuppressive medications or conditions. Additionally, all patients with cancer being treated with antiresorptive (AR) and/or antiangiogenic (AA) medications, regardless of concurrent immunosuppressive medications or conditions, are classified as high-risk groups.^58^

Understanding the distinction between these risk groups is crucial for the effective prevention and management of MRONJ. Beyond medication risk factors, several other important factors contribute to MRONJ, which will be briefly discussed in the following sections.

## Risk Factors for MRONJ

Since MRONJ was first discovered in 2003, awareness about it has grown, but there are still significant gaps in our understanding of its risk assessment, management, predictive factors, and treatment approaches.^25^

Current evidence suggests that several key risk factors contribute to the development of MRONJ, with the most critical factors listed in Table 2.^5,21,52^ A list of medications associated with MRONJ is provided in Table 3.^21,61^

**Table 2:** Most critical risk factors associated with MRONJ

	
Local risk factors	Tooth extraction Local infection and inflammation Inflammatory oral disorders Dentures Tooth abscesses Poor oral hygiene Dental implants
Pharmacological factors	Antiresorptive medications Antiangiogenic medications
Systemic factors	Malignant disease (cancer, multiple myloma, etc) Bone disease: (osteoporosis, Paget’s disease, etc) Diabetes Smoking


**Table 3:** Classification of the medications most commonly associated with MRONJ

	Medications	Mechanism of action	Example of generic or brand name
Antiresorptive medications	Bisphosphonates (Bps)	Synthetic pyrophosphate analogs that bind to hydroxyapatite, effectively inhibiting bone remodeling and metabolism.	Zoledronate Ibandronate Alendronate
Denosumab	Monoclonal antibody targeting receptor activator of nuclear factor kappa-B ligand (RANK-L), inhibits osteoclast maturation, survival, and function.	Xgeva Prolia
Antiangiogenic medications	Tyrosine kinase inhibitors (TKIs)	Block tyrosine kinase receptors involved in vascular endothelial growth factor (VEGF) signaling, crucial for angiogenesis.	Sunitinib Sorafenib
Monoclonal antibodies (mAb)	Bind to VEGF or its receptors, inhibiting angiogenesis.	Bevacizumab Aflibercept
Mammalian target of rapamycin (mTOR) inhibitors	Affect VEGF and platelet-derived growth factor (PDGF) production, exerting antiangiogenic effects.	Everolimus Temsirolimus


## Prevention

The increasing prevalence of cancer and osteometabolic diseases, along with the need for long-term treatment, make MRONJ prevention challenging.^11^ Therefore, preventive interventions should be implemented before, during, and after antiresorptive or antiangiogenic therapy to minimise MRONJ risks.^13,21,53^ Given MRONJ prevention complexities, a multidisciplinary approach that includes preventive measures with patient education and continuous monitoring is essential for effective care.

## Multidisciplinary Approach

A comprehensive approach involving dentists, primary care physicians, oncologists, oral and maxillofacial surgeons, and nurses is crucial for managing MRONJ effectively and improving patient symptoms and quality of life.^7,22,53^ This approach ensures continuous monitoring and individualised treatment plans tailored to each patient’s risk level. Regular dental evaluations and adherence to preventive measures, such as periodic periodontal screenings, are vital.^44^ Additionally, educational programs for healthcare providers and pharmacists are essential to enhance understanding and collaboration in managing MRONJ risks.^53,66^ Furthermore, educating patients is equally important, as proper oral hygiene and awareness of risks associated with certain dental procedures can significantly reduce the possibility of developing MRONJ.^57^

### a) Pre-treatment preventive measures

Before initiating AR or AA therapy, a comprehensive dental evaluation is necessary. Dentists should identify and treat any teeth with uncertain prognosis, focusing on reducing infection sources. This step is essential to ensure that dental procedures do not become risk factors for MRONJ once treatment begins.^7,35,59^

Invasive procedures such as extractions should ideally be completed 2–3 weeks before AR or AA therapy to allow healing.^59^ This aligns with guidelines promoting early dental intervention to minimise MRONJ risk, especially in high-risk patients.^35^

### b) In-treatment preventive measures

During AR or AA therapy, dentists must categorise procedures based on the patient’s risk level:

Contraindicated procedures: measures with a high risk of developing MRONJ that should be avoided unless necessary.
Possible procedures: measures with low risk of MRONJ but not preventive.
Recommended procedures: measures with low risk of MRONJ and considered preventive.^7^

Several techniques are used to minimise the risk of MRONJ during high-risk dental surgeries including tooth extractions, such as antimicrobial mouthwashes, antibiotic prophylaxis, and proper wound closure.^58^

### c) Post-treatment monitoring and management

After the completion of antiresorptive or antiangiogenic therapy, continuous monitoring of the patient’s oral health is crucial. Patients should be educated on reporting symptoms such as pain, swelling, or exposed bone, which enables prompt intervention and can prevent further complications. Long-term follow-up, especially for high-risk patients, is recommended to ensure that oral health is maintained even after cessation of therapy. Additionally, collaboration between the medical team ensures early detection and timely intervention.^35,59^

### d) Controversies and future directions

While bone turnover markers, such as C-terminal telopeptide (CTX), have been explored as potential predictors of MRONJ risk, they remain controversial due to inconsistent results in clinical studies. These markers have shown limited predictive value and are not currently recommended for routine use.^15^

Although no single technique can entirely eliminate MRONJ hazards, recognising risk factors and using tailored preventive measures for individual patients remains the most effective approach.^58,73^ Many existing recommendations about MRONJ are based on limited high-level evidence, requiring higher quality investigations for evidence-based treatments.^60^

## Primary Prevention

Primary prevention aims to prevent medical conditions before they occur, by focusing on maintaining good health, enhancing immune function, and reducing risk factors in individuals prone to disease.^38^ In dentistry, primary prevention focuses on preventing oral diseases, restoring oral health, and reducing local risk factors associated with various oral and dental diseases.^7^

Given that MRONJ is a severe side effect of certain medications, dental evaluation and treatment are necessary to be carried out before initiating AR and AA treatment.^45,65,66^ Therefore, it is advisable to postpone MRONJ-associated medications until after completion of invasive oral treatment like surgery or extraction of teeth with poor prognosis or those cannot be saved, and once proper post-surgical healing is ensured.^12,58^ Furthermore, dentists play a crucial role in primary prevention by identifying and minimising MRONJ risk factors through regular oral examinations, and professional dental cleanings.^13^ Encouraging patients to maintain good oral hygiene and scheduling regular check-ups every six months is another essential responsibility of dentists.^53^ Dietary modifications to reduce sugar intake are also recommended to prevent caries and subsequently minimise the need for extractions.^14^

To further reduce MRONJ risks, prompt diagnosis and treatment of local dental infections, particularly those affecting the bone, such as apical and marginal periodontal diseases, are critical.^53^ Therefore, patients starting bone-modifying agents should undergo a comprehensive dental evaluation, including radiographic assessment and MRONJ preventive measures.^22,53,58^ In addition, regular dental care, along with prophylaxis, caries control, conservative dentistry, and endodontic treatment, is crucial for maintaining oral health during the primary prevention phase.^58^ For patients using prosthetic dentures, it is recommended to perform pre-prosthetic surgery before initiating antiresorptive treatment to eliminate any stress points or sharp bone edges that may be present during denture use. The decision to fabricate a new denture, maintain the existing one, or perform invasive surgical procedures should be guided by the patient’s condition, specific needs, and medical history.^2^

Moreover, for cancer patients prone to bone metastases or needing chemotherapy, dental evaluation at the time of diagnosis and during chemotherapy for conditions such as oral mucositis is recommended.^53^ This ensures that any potential oral health issues are addressed rapidly, reducing the risk of complications.

## Secondary Prevention

Secondary prevention targets individuals who appear healthy but have early or mild stages of the disease, and focuses on an early diagnosis, when there are no obvious signs detectable during a physician’s visit.^38^ Assessment and clinical screenings are a common form of secondary prevention.^38^ In the case of MRONJ, early identification of sign and symptoms significantly improves management outcomes, emphasising the importance of secondary prevention.^13^

Secondary prevention of MRONJ is crucial across various dental specialties. Since patients are already at risk, dentists must employ conservative management strategies to prevent irritation to the bone, soft tissue, and blood supply. The recommended preventive measures across different dental specialties include the following:

### 1) Periodontology

According to recent studies, periodontal disease is a major risk factor for MRONJ. Therefore, in patients on AR and AA medicines, periodontal treatment is crucial to preventing and reducing this risk. However, periodontal surgery can also increase the risk of developing MRONJ. Consequently, non-surgical procedures such as root planing, scaling, and pocket probing are recommended (Table 4).^34^

**Table 4:** Secondary prevention for periodontal treatments in patients receiving AR and AA therapy

Risk level	Periodontological management approach
Low risk	No antibiotic prophylaxis needed in most cases, except for specific clinical scenarios
High risk	Prophylactic antibiotics recommended: Amoxicillin 1 g three times daily (or combined with clavulanic acid/metronidazole) Antibiotic treatment starts 2-3 days before intervention and continues for 5-17 days postoperatively, depending on healing progress and risk factors


#### a) Periodontal therapy in low-risk patients

Patients at low risk typically do not require antibiotic prophylaxis. Exceptions may occur in specific clinical situations in which the patient has concurrent infections or other risks.^39^

#### b) Periodontal therapy in high-risk patients

Patients at high risk should receive prophylactic antibiotics before periodontal interventions, regardless of the duration of therapy. The standard antibiotic regimen recommendation is amoxicillin 1 g three times daily. In cases where broader coverage is needed, this can be combined with clavulanic acid, or with 500 mg of metronidazole twice daily.^39^

Prophylaxis typically starts 2-3 days before the periodontal intervention. However, there is no consensus on the postoperative administration period, so the dentist must determine the duration, typically ranging from 5 to 17 days, based on the patient’s risk factors and healing progress.^39^

### 2) Endodontics

In patients receiving AR and AA therapy, endodontic treatment plays a key role in secondary prevention of MRONJ, providing a safer alternative to extractions by addressing infections and periapical lesions while minimising invasive interventions (Table 5).

**Table 5:** Secondary prevention for endodontic treatment in patients receiving AR and AA therapy

Category	Key considerations
Low-risk patients	Root canal treatment is preferred as it eliminates infections without increasing MRONJ risk by invasive treatment intervention.
Apicoectomy should generally be avoided due to similar risks as tooth extraction.
Endodontic treatment helps resolve periapical lesions and reduces infection-related complications.
High-risk patients and precautions to enhance safety and efficacy	Non-surgical root canal therapy is recommended over invasive treatments to minimize MRONJ risk associated with extractions.
1. Informed consent	Obtain consent and coordinate care with the patient’s treating physician.
2. Pre-procedure rinse	Use a chlorhexidine mouthwash for 1 min to reduce microbial load and potential bacteremia risk.
3. Avoid vasoconstrictors	Select anesthetics without vasoconstrictors.
4. Minimise trauma	Place rubber-dam and limit soft tissue trauma during its application.
5. Disinfection	Disinfect the tooth and rubber-dam with a 5% tincture of iodine.
6. Apical management	Use electronic apex locators to avoid apical extrusion of debris and maintain apical constriction.
7. Debris control	Use NiTi rotary systems for shaping to reduce debris extrusion. Avoid reciprocating systems.
8. Obturation	Use cold lateral compaction. Avoid warm gutta-percha obturation technique. Avoid overfilling or overextension.


#### a) Endodontic treatment in low-risk patients

Endodontic treatment is not considered a risk factor for MRONJ; rather, it can help prevent MRONJ by addressing periapical lesions that are associated with infection. Nevertheless, apicoectomy carries similar risks as tooth extraction and should generally be avoided. Thus, root canal treatment is preferred for low-risk patients on antiresorptive agents, as it resolves periapical lesions without exacerbating the risk of MRONJ.^34^

#### b) Endodontic treatment in high-risk patients

To minimise this risk of developing MRONJ after tooth extraction in high-risk patients, non-surgical root canal therapy is recommended as an alternative, since it reduces infection and prevents it from spreading to the periapical area. To ensure the safety and efficacy of endodontic procedures in these patients, the following precautions should be observed:^3^

Informed consent: before starting treatment, providing information about the risk level of MRONJ to the patient, obtaining informed consent and coordinating with the patient’s treating physician are essential steps to ensure comprehensive care.^58^
Pre-procedure antimicrobial rinse: a chlorhexidine mouthwash rinse should be administered for 1 min to reduce the oral microbial load, thereby decreasing the potential risk of bacteremia during the procedure.^3^
Avoiding vasoconstrictors: to prevent compromised vascularisation, anesthetic agents containing vasoconstrictors should be avoided, as the anti-angiogenic properties of drugs such as bisphosphonate may already impair blood supply.^3^
Minimising trauma: In order to maintain a fully aseptic environment and minimise stress, particularly to the soft tissues during rubber-dam application, efforts should be executed.^3^
Disinfection: for further reduction of the infection risk, the tooth and rubber-dam should be disinfected with an appropriate agent, such as tincture of iodine (5%).^3^
Debris control and apical management: careful management of the apical foramen is crucial to avoid bacteremia from extruded debris. To accurately maintain apical constriction and prevent apical extrusion, electronic apex locators should be used. Nickel-titanium (NiTi) rotary systems are recommended for root canal shaping to limit debris extrusion, while reciprocating systems should be avoided.^3^
Obturation: When performing obturation, precise techniques should be employed to avoid overfilling and over-extrusion; cold lateral compaction is preferred over warm obturation techniques to effectively manage apical filling and reduce the risk of apical overfilling.^3^

### 3) Prosthodontics

The primary objective of prosthetic treatment for MRONJ patients is to minimise stress load and impact of the dental prostheses on the supporting mucosa, especially in patients with cancer-related bone loss, which makes the ridge more susceptible to functional loads.^2^ This treatment approach varies based on whether patients are considered low or high risk for MRONJ (Table 6).

**Table 6:** Secondary prevention for prosthodontic treatment in patients receiving AR and AA therapy

Category	Low-risk patients	High-risk patients
Removable prosthesis	Telescopic crowns recommended for rigid stabilisation and enhanced retention.	Telescopic crowns to reduce mucosal irritation and MRONJ risk, especially compared to clasps.
Heat-cured resilient liners or heat-activated acrylic resin reduce pressure points on the mucosa.	Heat-cured liners or resins used for even load distribution.
Denture base thickness of 1.5–3 mm ensures even load distribution.	Maintain 1.5–3 mm denture base thickness.
Meticulous cleaning with 5.25% sodium hypochlorite is mandatory.
Regular cleaning with a 5.25% sodium hypochlorite solution to reduce contamination.	Frequent relining required to manage occlusal force distribution and prevent MRONJ.
Patients should remove dentures for at least 12 hours daily and undergo evaluation every 2–3 months.
Fixed prosthesis	Supragingival margin preparation facilitates hygiene and protects gingival tissues.	Supragingival margin preparation reduces bacterial invasion risk and facilitates oral hygiene.
Routine antibiotic prophylaxis not necessary; while chlorhexidine mouth rinse can reduce bacterial load during impression procedures.	Prophylactic antibiotics and chlorhexidine mouth rinses are recommended during tooth preparation and impression taking.


#### a) Prosthodontic treatment in low-risk patients

Removable prostheses: For low-risk patients, removable prostheses can generally be managed without extensive modifications. However, attention should still be given to minimising the potential of trauma to the mucosa.^2,27^
Fixed prostheses: Fixed prostheses for low-risk patients carry a minimal risk for MRONJ. Nevertheless, supragingival finish line preparation is advisable to facilitate easier hygiene and protect the gingival tissue. Routine antibiotic prophylaxis may not be necessary for low-risk patients, but a chlorhexidine mouthrinse could help reduce bacterial load during impression procedures.^2^

#### b) Prosthodontic treatment in high-risk patients

i) Removable prostheses: For high-risk patients, more stringent preventive measures are indicated:

Telescopic crowns remain crucial for rigid stabilisation and minimising mobility. In high-risk patients, this can significantly reduces mucosal irritation and the chance of developing MRONJ compared to removable partial dentures with clasps.^2,27^
Denture bases should be carefully prepare and fabricated, with heat-cured resilient liners or heat-activated acrylic resins used to ensure even load distribution. Based on texture and firmness, frequent replacement of definitive soft liners is advised, as uneven forces could trigger MRONJ.^2^
Denture base thickness should still be maintained at 1.5–3 mm, and meticulous cleaning practices with a 5.25% sodium hypochlorite solution should be followed to avoid infections.
As the second most frequent causes of MRONJ are sore spots and ill-fitting dentures, denture supporting tissue should undergo clinical and radiographic evaluation every two to three months.^2^
Patients should remove their dentures for at least 12 hours daily.^2^
At the time of denture delivery, meticulous finishing and polishing should be performed to round and smooth all denture borders, especially in the mandible. To protect the oral mucosa from damage during placement, areas of high pressure should be identified and relieved using a pressure-indicating paste. Sore spots and the risk of MRONJ are possible side effects of newly placed dentures, especially within the first three weeks. These potential risks should be explained to patients, and they should be encouraged to see their dentist immediately if any sore spots develop. If a prompt consultation is not feasible, the dentures should not be worn until the problem is resolved.^2^

For patients with active MRONJ, conservative prosthetic management is advised to improve their quality of life. This approach minimises pain and discomfort, optimises function, and prevents secondary infections by ensuring the denture is appropriately fitted and kept clean.^2^

ii) Fixed prosthesis: Fixed partial dentures exhibit a lower risk of MRONJ compared to mucosa-supported removable dentures, but preventive measures should still be implemented:

Supragingival finish line preparation is recommended to reduce the risk of bacterial invasion and protect gingival tissues, thus facilitating oral hygiene.
Prophylactic antibiotics and chlorhexidine mouth rinses are typically recommended during tooth preparation and impression taking in high-risk patients to prevent bacterial contamination and manage the increased risk of MRONJ.^2^

For all high-risk patients, communication with the treating physician, informed consent, and careful treatment planning are essential to minimising complications.

### 4) Orthodontics

Orthodontic treatment has significantly expanded from pediatric patients to adults, presenting new challenges for orthodontists. These challenges involve managing the effects of multiple medications taken by adult patients, complex health conditions, and altered physiological states. Consequently, orthodontic therapy can greatly enhance a patient’s quality of life, and at-risk individuals should not be excluded from its benefits.^74^

Importantly, in clinical settings, there is no evidence to support a correlation between orthodontic treatment and a higher risk of developing MRONJ. Although patients on antiresorptive medications can initiate orthodontic treatment, achieving optimal tooth movement may be challenging because of these medications. However, caution is advised when treating these patients.^34^

Orthodontists must thoroughly investigate risk factors, focus on up-to-date evidence, and collaborate with other medical professionals to determine the most effective treatment approach, and to minimise complexities.^74^

According to a case report of active bone surgery followed by endocrine evaluation, which demonstrates that successful surgical-orthodontic treatment is practicable in adult patients with osteoporosis and normal bone turnover.^37^ Orthodontic treatment, while generally considered safe, requires special consideration in patients at risk of the MRONJ.

#### a) Orthodontic treatment in low-risk patients

For patients using low-dose antiresorptive drugs, which can slow down tooth movement, the risk of MRONJ is generally low, making orthodontic treatment viable with caution. It is crucial to avoid invasive procedures, such as extractions, and maintain good oral hygiene. However regular monitoring during orthodontic treatment is crucial to detect early signs of MRONJ.^74^

#### b) Orthodontic treatment in high-risk patients

For this group of patients on high-dose antiresorptive or antiangiogenic drugs undergoing orthodontic treatment, careful treatment planning and preventive strategies are essential. Orthodontic movement that may strain bone should be minimised, as high-risk patients are especially vulnerable to MRONJ following invasive procedures.^34,74^ Non-invasive orthodontic approaches are preferred over more invasive options whenever feasible. However, if tooth extractions or surgical interventions are needed, alternative, minimally invasive solutions should be considered. Close coordination with the patient’s medical team is crucial for assessing and adjusting medication timing to reduce MRONJ risk.^34,74^

Nevertheless, there have been cases where orthodontists were not informed about patients’ treatments with high-risk medications.^74^ This highlights the importance of comprehensive patient histories and inter-professional communication to ensure safe and effective orthodontic treatment.

### 5) Dental implants

MRONJ has been observed in patients with osteoporosis or cancer who have taken oral bisphosphonate, particularly following dental implant placement, ex-implant procedures, and in case of peri-implantitis.^34^

Therefore, highly stringent care regimens are strongly advised. These regimens should include regular follow-up intervals, radiographic examinations, regular dental hygiene, and cleaning of the tissue surrounding dental implants in the dental office. Additionally, regular oral assessments of the soft and hard tissues surrounding teeth and implants are essential.^34^

#### a) Dental implants in low-risk patients

For patients in this group, such as those with osteoporosis who have used oral bisphosphonates for less than three years without additional risk factors, dental implants may be viable with precautions.^74^

Prevention protocols:

Detailed medical history and consultation with the patient’s physician are essential to determine the duration and dosage of antiresorptive medication.^34^
Chlorhexidine rinses prior to surgery can reduce oral microbial load and prevent infection, lowering MRONJ risks.^74^
Implant placement should be minimally invasive to protect the bone and soft tissues.^37^
Careful monitoring after implant placement surgery, including regular clinical and radiographic evaluations, is essential to identify early signs of bone necrosis or implant failure.^37^

#### b) Dental implants in high-risk patients

For high-risk patients, such as cancer patients receiving high-dose antiresorptive medications, implant-related surgical procedures are generally contraindicated. These patients face a significantly higher risk of MRONJ, and alternative treatments should be considered. If implants are necessary, such as for prosthodontic rehabilitation, a thorough risk-benefit analysis is required in consultation with the oncologist and other healthcare providers.^34^

### 6) Dental surgery

Dental infections are significantly related to MRONJ, making the prompt identification and treatment of an underlying infection crucial for preventing the condition from developing.^53^ Managing infections helps to avoid MRONJ, which is associated with an increased risk of MRONJ. However, when the infected tooth is not restorable, extraction may become necessary. In such cases, minimising trauma during the extraction and following the prevention protocols is essential to reduce the risk of MRONJ (Table 7).^53^

**Table 7:** Secondary prevention for tooth extraction in patients receiving AR and AA therapy

Aspect	Low-risk patients	High-risk patients
Antibiotic prophylaxis	Generally not required unless specific clinical factors indicate otherwise.	Recommended starting three days before surgery: amoxicillin (3 g/day) or clindamycin (1800 mg/day) for patients allergic to penicillin.
Pre-surgical care	Dental hygiene scaling one week before surgery. Chlorhexidine rinses twice daily before the procedure. No drug hiatus recommended to prevent osteoporosis-related fractures.	Dental hygiene scaling one week before surgery. Chlorhexidine rinses twice daily before the procedure. Pre-operative radiographs to identify potential MRONJ risk factors and aid surgical planning.
Surgical techniques	Gentle surgical approach to minimize trauma. Ensure complete soft tissue closure after extractions.	Advanced surgical techniques to minimize complications: Flap elevation: Mucoperiosteal flap for visibility with minimal trauma. Alveoloplasty: Smooth sharp bone edges to reduce irritation and enhance healing. Tension-free suturing: Promotes soft tissue healing. Platelet-rich fibrin (PRF): Enhances angiogenesis and supports soft tissue and bone healing.
Post-surgical care	Follow standard post-surgical protocols for monitoring healing.	Chlorhexidine mouthwash (0.12%) twice daily. Antibiotic administration.
Follow-up care	Routine follow-ups based on standard dental practice.	Monitor healing closely. Schedule regular radiographic evaluations to ensure proper bone regeneration and early detection of MRONJ signs.


#### a) Tooth extraction in low-risk patients

Patients with osteoporosis taking oral bisphosphonate or denosumab for less than three years, and with no other major risk factors (such as corticosteroid use), can generally undergo dental extractions without major modifications to their treatment plan.^58^

Antibiotic prophylaxis is generally not recommended before surgery, except in specific clinical situations.^39^

Pre-surgical care involves dental hygiene scaling one week prior to surgery and chlorhexidine rinses twice daily.^71^

A hiatus from taking medications is generally not recommended for these patients, as discontinuing medication may increase the risk of osteoporosis-related fractures.^9^

However, the focus is on careful surgical technique, minimising trauma, and ensuring proper soft tissue closure after the procedure.^43^

#### b) Tooth extraction in high-risk patients

For high-risk patients (such as cancer patients on high-dose AR/AA therapy or those with osteoporosis on AR/AA medication more than three years), preventive measures are more comprehensive and include the following steps.

Antibiotic prophylaxis:^71^ Antibiotic administration for MRONJ is largely empirical, particularly the timing of initiation in the preoperative phase of tooth extraction, duration of postoperative continuation, as well as choice of agents, dosage, or route of administration.^8^ A total duration of 7–14 days is commonly reported in the literature;^8^ however, the length of postoperative continuation may vary depending on how early antibiotics are initiated before surgery, and continuing them afterward to complete the course, often until soft tissue healing is achieved, although this practice varies across clinical settings. For example, Di Fede et al^21^ and Pick et al^54^ suggest starting antibiotics one day prior to surgery. Furthermore, Di Fede et al^21^ suggest continuing the antibiotics for a minimum of 6 days after the procedure. Despite this variability, certain antibiotics such as penicillin, amoxicillin/clavulanic acid, metronidazole, or combinations of these, are more frequently prescribed in management of MRONJ.^8,10^ The Italian Societies of Oral Pathology and Medicine and Maxillofacial Surgery (SIPMO-SICMF) has recommended penicillin combined with metronidazole as first-line treatment, administered orally in outpatients and intravenously in hospitalised patients, typically for one to two weeks.^8^ In cases where standard treatment fails or the patient is allergic to penicillin/cephalosporins, other antibiotics such as erythromycin, clindamycin, or ciprofloxacin may be considered.^8^ Although clindamycin is still listed as a potential option in MRONJ protocols, recent guidelines for infective endocarditis prophylaxis have excluded it due to concerns over its lower efficacy and higher risk of adverse effects.^18,70^

Pre-surgical care: similar to low-risk patients, dental hygiene scaling one week prior to surgery and chlorhexidine rinses twice daily is advised.^71^ Additionally, in designing a preventive plan, identifying the pre-operative radiographic examination is required.^49^

Mucoperiosteal flap elevation: careful elevation of the mucoperiosteal flap helps ensure proper visibility and access during the extraction while minimising trauma to soft tissue and blood supply.^22,35^
Alveoloplasty: smoothing sharp bone edges helps reduce the risk of irritation and enhances wound healing after surgery.^22,35^ Since performing alveoloplasty has been shown to significantly reduce necrotic bone formation and improve outcomes in high-risk patients, it is particularly recommended in patients at elevated risk for MRONJ.^55^
Tension-free suturing: suturing should be performed without exerting tension on the soft tissue, as tension can impede healing and increase the risk of complications.^22,35,43^
Platelet-Rich Fibrin (PRF): the use of platelet-rich fibrin (PRF) during dental extractions has emerged as an effective preventive measure against MRONJ. PRF enhances wound healing by promoting angiogenesis and local immunomodulation, which supports soft tissue healing and bone regeneration. Studies report positive outcomes of using PRF in patients at risk of MRONJ, with good soft tissue healing and minimal post-surgical complications.^4,5,48^
Systemic erythropoietin (EPO): Research on systemic erythropoietin (EPO) administration before and after extractions in zoledronic acid-treated rats suggests potential benefits, such as reducing inflammation, promoting angiogenesis, and supporting bone healing, though no significant improvement in overall bone regeneration was observed.^23^

Post-surgical care: After surgery, regular follow-up appointments are essential to monitor the healing process and ensure early detection of any signs of MRONJ, including clinical and radiographic evaluations.^59^ Regular follow-up visits should be scheduled after surgery in high-risk patients with a specific focus on soft tissue healing and bone regeneration, and to monitor for early signs of MRONJ. Studies suggest that extended monitoring period is crucial, as MRONJ can present weeks or even months after surgery.^59^ Wound care includes the use of diluted 0.12% chlorhexidine mouthwash twice daily to keep the surgical area clean and reduce bacterial load.^56^

Signs of MRONJ recurrence: Patients should be educated on the sign and symptoms of MRONJ, such as localised pain, exposed bone, infection, and swelling. Early detection of these symptoms is critical for timely intervention. Studies emphasise the importance of patient education in reducing the risk of MRONJ progression.^68^

By adhering to these post-surgical protocols, dentists can help to minimise the MRONJ risk and support optimal healing for the patients.

##### Staging

The MRONJ staging system, which includes five stages, is determined by radiographic and clinical evaluations. It was introduced in 2006 by Ruggiero et al^59^ and selected by the AAOMS in 2007, with an update in 2014.

When determining the most appropriate treatment for each patient, the staging of MRONJ is crucial.^47^

According to the AAOMS, MRONJ lesions are divided into five categories:^42,58^

In at-risk patients, necrotic bone or other symptoms of MRONJ are not observed. In this stage, active treatment is not indicated.
Stage 0 (non-exposed bone): extracted tooth area lacks the clinical manifestations of bone destruction, but instead shows poorly defined indicators or symptoms on radiographs. Clinical examination may reveal unexplained pain, sinus pain, and pain spreading to the temporomandibular joint (TMJ), along with mobile teeth without a history of periodontal issues or swelling. Radiographic examination may show sclerotic bone alterations and lack of new bone formation in recently extracted tooth sockets.
Stage 1: while radiographic findings are similar to stage 0, clinically they may represent exposed bone or fistula, although it is painless and does not show signs of infection.
Stage 2: radiographically presented as in previous stages, with clinical symptoms as in stage 1, but differing from that by presenting inflammation and/or infection.
Stage 3: not only presents symptoms of previous stages, but also exhibits at least one of the following clinical manifestations: fistula that can be probed extraoraly, pathological fracture, oroantral communication, necrosis of bone extended over the alveolar bone to the surrounding areas such as the mandibular ramus, inferior border of the mandible or zygoma, and sinus floor in maxilla presenting osteolysis.

## Tertiary Prevention

Tertiary prevention focuses on managing the medical and consequent phases of a disease, aiming to reduce its severity and associated consequences in symptomatic patients.^38^ Unlike secondary prevention, which aims to prevent the disease onset, tertiary prevention addresses conditions that have already developed, often involving rehabilitation measures.^38^

In diagnosing MRONJ, radiographic examinations are crucial in identifying early stages through radiographic features such as sclerotic bone, and poorly defined radiolucency.^1^ In the advanced stages, developed sequestrum, enlargement of the lamina dura, and pathological fractures may resemble the radiological appearance of chronic osteomyelitis.^1^ Digital imaging modalities like CT and CBCT reveal MRONJ lesions characteristics, highlighting bone sclerosis, bone loss, cortical deterioration, periosteal changes, and fistulas.^1^ Since MRI has historically been considered less accurate for skeletal examination, the appearance of ONJ on MRI has been viewed as uncertain and unpredictable.^1^ However, recent studies have demonstrated MRI’s increasing value in the evaluation of MRONJ. Specifically, ultrashort echo time (UTE) sequences and dynamic contrast-enhanced (DCE) MRI protocols have shown potential in improving the detection of bone destruction and marrow abnormalities.^31,64^ As MRI techniques such as UTE and DCE continue to progress, MRI’s role in evaluating osseous changes in MRONJ may expand even further.

In this phase, the primary objective of therapy is to prevent MRONJ from progressing to a more severe stage, reduce bone necrosis incidence, improve patient’s quality of life, manage symptoms, infection, and particularly pain.^26,26^ In systemic management of MRONJ, antibiotics are the most common and first prescription line, including penicillin, metronidazole, and amoxicillin.^51^

Although initial conservative therapy involves antibiotics, antimicrobials, and analgesics, recent studies underscores the benefit of early surgical intervention to ensure complete necrotic bone removal,^71^ while good oral hygiene should be maintained alongside both conservative and surgical therapies.

Nevertheless, dentists must apply the most recent data to assess the risk, recognise the symptoms at the earliest, and administer appropriate treatments based on urgency and patient condition.^69^ A collaborative decision-making approach ensures tailored nonoperative and operative care across disease stages.^58^

### Non-invasive or Non-operative Procedures

Patients in MRONJ Stages 0 and 1 may benefit from therapeutic interventions such as antimicrobial rinses (e.g., chlorhexidine) and systemic antibiotics; in Stages 2 and 3, these measures may be used as adjuvant therapy. In later phases necessitating surgical intervention, however, conservative medicinal treatment might be more beneficial, particularly if the patient rejects surgery or is unable to undergo surgery due to health conditions.^51^

However, chlorhexidine’s efficacy as a mouthwash remains contentious, with mechanical wound care showing promising adjunctive benefits in reducing disease persistence and discomfort.^32^

Alternative therapies such as ozone therapy and hyperbaric oxygen lack substantial evidence for MRONJ treatment and are not currently recommended.^58^ Research on Vitamin E, pentoxifylline, and teriparatide continues to explore their potential preventive and therapeutic roles.^58^

### Invasive or Operative Procedures

The most important approach for treating MRONJ’s advanced phases is surgery, which is successful in phases 2 and 3.^51^ According to On et al,^51^ surgical intervention even in the early stage of MRONJ has a higher success rate, offering a short course of treatment while minimising pain, controlling infection, relieving soft tissue, and reducing bone loss.

Curettage and surgical debridement are effective for lesion removal in stages 1 and 2,^32^ while partial osteonecrotomy serve as an effective treatment to manage MRONJ in all stages including stage 1, ensuring clear boundaries beyond necrotic margins.^58^

Under local anesthesia, surgical procedures involve meticulous removal of necrotic bone, preserving cortical plates with full-thickness mucoperiosteal flaps.^71^ After extracting the teeth causing the necrotic area, thorough bone curettage and osteoplasty ensure viable white bone and optimal blood flow, and primary flap closure using absorbable sutures.^71^

Following the operational period and eventually isolated bone sequestration in MRONJ patients, a preliminary pharmacological phase is based on antibiotics, antiseptics, and analgesic medications.^71^ Virtual Surgical Planning (VSP) enhances precise delineation of MRONJ lesions, employing custom CAD/CAM materials to reduce surgical duration and ensure complete necrotic bone resection, potentially preventing mandibular fractures and reducing morbidity.^16^ Clinical outcomes are generally favorable, though further research is needed.^16^

Since ONJ can manifest years after surgery, patients should be monitored closely.^19^

### Indications for Non-surgical vs Surgical Treatment in Patients with Developed MRONJ

Treatment decisions for MRONJ should be based on different factors, including the stage of the disease, the patient’s overall health, and the potential risks and benefits of surgery. For patients in the early-stage of MRONJ, those with significant comorbidities or palliative care needs, non-surgical treatment is often suitable. On the contrary, patients with advanced stages of MRONJ, or those whose conservative treatment has failed, and those in medically stable conditions are typically candidates for surgical intervention. To achieve optimal results in MRONJ management, a customised multidisciplinary strategy is essential.^20,24^

**1. Non-surgical (non-invasive) treatment** ^13,20,58^

In the early stages of MRONJ or in patients with major comorbidities, where the risks of surgery may outweigh the benefits, non-surgical or conservative therapy is typically recommended. The goals of non-surgical treatment are to lessen symptoms, stop the disease from becoming more severe, and enhance quality of life.^13,20^

Early stages of MRONJ (stages 0 and 1): conservative management is frequently the main option for patients in the early stages of MRONJ who report with minimal bone exposure or no exposed necrotic bone. Analgesics, antimicrobial mouthwashes (such as chlorhexidine), and systemic antibiotics are among the treatment protocols. According to recent research, conservative treatment can be beneficial, particularly when associated with good oral hygiene, since it may postpone the need for surgery and stop the condition from getting worse. Surgical intervention may still be considered.^13,20,58^
Patients with significant comorbidities: for patients with uncontrolled systemic illnesses, such as severe cardiovascular disease, uncontrolled diabetes, patients receiving chemotherapy or with other immunosuppressive conditions that impair their ability to recover, non-surgical care is recommended. These patients are at high risk of postoperative complications, such as infections and poor wound healing, making conservative approaches the first line of treatment.^13,20,58^
Patients with limited life expectancy or palliative care needs: invasive treatments intended for long-term disease control might not prove beneficial for patients with advanced systemic disorders, such as end-stage cancer or severe organ failure. In these situations, the priority shifts toward palliative care, which focuses on infection prevention and controlling pain. These groups of patients are usually treated with antibiotics, painkillers, and minimal surgery when needed.^13,20,58^
Patient refusal or surgical contraindications: some patients may refuse surgery for personal reasons, including fear of complications or prior negative surgical experiences. Additionally, some patients may refuse it for medical reasons. For instance, patients unable to tolerate anesthesia or those contraindicated for surgery due to blood disorders or medications (e.g., anticoagulants) are often managed non-surgically.^13,20,58^

**2. Patients Indicated for Surgical (Invasive) Treatment** ^13,20,58^

Invasive or surgical interventions are typically indicated for patients in more advanced stages of MRONJ or for those who fail to respond to conservative treatment. Surgery aims to remove necrotic bone, control infection, and prevent further complications such as pathological fractures.^13,20,58^

Advanced stages of MRONJ (stages 2 and 3): patients with exposed necrotic bone, infection, and inflammation, particularly those in Stage 2 and Stage 3, are often candidates for surgical intervention. Surgery may involve debridement, curettage, or more extensive procedures like osteonecrotomy. Early surgical intervention, even in Stage 1, has been shown to improve outcomes, reduce infection risk, and alleviate symptoms more effectively than conservative treatment alone. Surgical interventions in early-stage MRONJ have been associated with a higher success rate, faster recovery, and better pain control.^13,20,58^
Patients who did not respond to conservative treatment: surgery or invasive treatment is indicated if non-surgical treatments were not curing an earlier stage of MRONJ. Persistent or worsening symptoms, such as uncontrolled infection, increasing pain, or further bone necrosis, necessitate surgical removal of the necrotic tissue to prevent progression. Patients who fail conservative management often have improved outcomes after undergoing surgical debridement.^13,20,58^
Medically stable patients: patients without significant comorbidities or those with well-managed chronic conditions (e.g., controlled diabetes or hypertension) are typically good candidates for surgery. Surgical interventions, including osteonecrotomy, are safe and effective when the patient’s general health allows for appropriate wound healing and postoperative recovery.^13,20,58^

### Quaternary Prevention

Quaternary prevention in general practice is designed to identify individuals at risk of overmedicalisation, protect them from unnecessary medical interventions, and recommend appropriate measures.^38^ It was introduced by Marc Jamoulle, as described by Kisling and Das,^38^ and the concept specifically targets patients who are ill but not diseased, aiming to prevent medical procedures that could potentially cause more harm than benefit.

However, the application of quaternary prevention in the context of MRONJ lacks sufficient study. Further research is essential to define and express unnecessary interventions, and appropriate strategies to prevent the development of underlying risk factors of MRONJ like osteoporosis and cancer.

## MATERIALS AND METHODS

A comprehensive literature review was conducted using multiple databases, including Google Scholar and PubMed, to collect relevant studies. The search employed a combination of Boolean operators (AND, OR) with keywords included “medication-related osteonecrosis of the jaw”, “MRONJ”, “antiresorptive medication”, “antiangiogenic medication”, “MRONJ and periodontics”, “MRONJ and prosthodontics”, “MRONJ and orthodontics”, “MRONJ and implantology”, “MRONJ and dental surgery”, “MRONJ and endodontics”, and prevention measures like “primary,” “secondary,” “tertiary,” and “quaternary prevention of MRONJ”. The search was limited to English articles published between 2007 and 2024.

Initial searches yielded 121 articles, which were then screened for relevance based on titles and abstracts. Articles were included if they focused on MRONJ prevention, diagnosis, risk factors, and management strategies, with an emphasis on prevention in various branches of dentistry. Studies that did not directly address these areas. Duplicate entries were removed.

The data extracted from the final selection of articles included primary findings, prevention measures, and clinical outcomes. These were synthesised qualitatively to identify common trends, gaps in the literature, and consensus on prevention strategies.

To enhance clarity and coherence, OpenAI’s ChatGPT-4o, 4o mini, and 3.5 models were used to provide suggestions and corrections, with QuillBot assisting in paraphrasing where necessary. These tools were utilised to refine the language and ensure flow but were not involved in generating original scientific content. The final text underwent a thorough manual review to ensure academic integrity.

## RESULTS

MRONJ is primarily linked to drugs referred to as antiresorptive or antiangiogenic medicines, which are known to inhibit the generation of new blood vessels, slow mucosal healing, or affect bone resorption, even though the precise pathophysiology is still unknown.^21,41^ MRONJ development following tooth extraction is influenced by local and systemic factors such as local infection, inflammation, treatment with AR and AA drugs, oral diseases, ill-fitting dentures, tooth abscesses, inadequate oral hygiene, and dental implants.^5,6^ To recognise early signs of MRONJ, such as sclerotic bone and poorly defined radiolucency, panoramic radiographs are required, while in advanced stages CT and CBCT are indicated.^1^

### Prevention Measures as Management Strategies

Prevention strategies are typically categorised into four main groups, outlined in Table 8.^38^ Current knowledge regarding MRONJ prevention is well-documented for the first three categories, but understanding remains limited in quaternary prevention.

**Table 8:** Definitions of prevention categories

Prevention	Definition
Primary	Involves proactive measures targeting communities or individuals at risk, aiming to prevent the onset of medical diseases before they occur.
Secondary	Prioritizes early diagnosis during routine appointments with a physician, aiming to identify mild signs of illness in seemingly healthy individuals before visible symptoms appear.
Tertiary	The objective is to manage both the symptomatic stages of a disease and its related effects, aiming to minimize the severity of the illness and its subsequent impacts on patients.
Quaternary	It is known as a preventive measure aimed at identifying individuals susceptible to overmedicalization, thereby safeguarding patients from unnecessary medical interventions and recommending morally appropriate actions for their health.

Primary prevention of MRONJ: dentists play a crucial role in primary prevention by promoting proper oral hygiene, regular check-ups, and cleanings. This reduces MRONJ risk factors and helps identify early symptoms.^13,53^ Patients starting treatment with bone-modifying agents should have radiographic evaluation, diagnosis, and treatment of local oral infections, particularly those impacting the bone, such as endodontic and periodontal diseases.^22,53,58^
Secondary prevention of MRONJ: secondary prevention focuses on minimising the trauma of dental extractions or other surgeries to reduce MRONJ risk.^53^ Key measures include minimal trauma techniques, elevating the mucoperiosteal flap, careful removal of sharp bone edges, and tension-free suturing during extractions.^22,35^ Additionally, platelet-rich fibrin (PRF) application during tooth extraction is recommended to promote healing and further reduce MRONJ risk.^48^
Tertiary Prevention of MRONJ: tertiary prevention addresses patients who have already developed MRONJ. Treatment is tailored to the stage of MRONJ, as outlined by the American Association of Oral and Maxillofacial Surgeons (AAOMS). Invasive or non-invasive interventions are chosen depending on disease severity, ranging from antimicrobial therapy to surgical debridement and resection.^38,58^
Quaternary prevention of MRONJ: the goal of quaternary prevention is to reduce the unnecessary medicalisation of patients by lowering the prevalence of osteoporosis and cancer, thereby decreasing the need for AR and AA medications. More research is necessary to understand the role of quaternary prevention in MRONJ and how best to avoid unnecessary interventions.

## DISCUSSION

Although MRONJ has been linked to drugs used to treat cancer and osteoporosis, the exact pathophysiology and mechanisms causing osteonecrosis to remain unclear. This ambiguity makes it more difficult to establish effective preventive interventions. Therefore, further research and experimental studies are needed to identify the underlying causes of MRONJ and more specialised preventive and management strategies.

MRONJ is primarily associated with antiresorptive or antiangiogenic medicines, which inhibit blood vessel generation, slow mucosal healing, and affect bone resorption, although their exact pathophysiology remains unknown.^21,41^

Current preventive measures in clinical practice aim to reduce the risk of MRONJ onset and progression. One promising intervention highlighted in recent literature is the application of PRF during tooth extraction procedures. PRF, known for its ability to enhance wound healing and tissue regeneration, shows potential as a cost-effective adjunctive therapy in reducing the incidence of MRONJ.^48^ However, further investigation in larger, more diverse patient cohorts are necessitated to validate its efficacy definitively.

## CONCLUSION

MRONJ is a rare but serious condition, especially in patients undergoing antiresorptive or antiangiogenic therapies. However, with the application of proper preventive measures, it can be prevented or its severity may be reduced. This review provides a comprehensive overview of current knowledge in different specialties in dentistry, emphasising the importance of ongoing research and a multidisciplinary approach to improve patient quality of life. Moreover, the exact etiology of MRONJ is unknown and as a result, lacks proven effective prevention, and further research and experiments are indicated.

## Abbreviations

American Association of Oral and Maxillofacial Surgeons (AAOMS)

Antiangiogenic (AA)

Antiresorptive (AR)

Bisphosphonates (BPs)

Bone-modifying agents (BMA)

C-terminal telopeptide (CTX)

Computed tomography (CT)

Computer-Aided Design/Computer-Aided Manufacturing (CAD/CAM)

Cone-beam computed tomography (CBCT)

Dynamic contrast-enhanced (DCE)

Intramuscular (i.m.)

Intravenous (i.v.)

Intravenous (IV)

Italian Societies of Oral Pathology and Medicine and Maxillofacial Surgery (SIPMO-SICMF)

Mammalian target of Rapamycin (mTOR)

Medication-related osteonecrosis of the jaw (MRONJ)

Monoclonal antibodies (mAb)

Nickel Titanium (NiTi)

Osteonecrosis of the jaw (ONJ)

Osteoradionecrosis (ORN),

Platelet-derived growth factors (PDGF)

Platelet-Rich Fibrin (PRF)

Receptor activator of nuclear factor kappa-B ligand (RANK-L)

Temporomandibular jont (TMJ)

Tyrosine kinase inhibitors (TKIs)

Ultrashort echo time (UTE)

Vascular endothelial growth factor (VEGF)

Virtual Surgical Planning (VSP)
